# Poststroke Neurorehabilitation Using a Soft Robotic Glove Combined With a Virtual Environment: Preliminary Study on Feasibility, Safety, Effects, and User Satisfaction

**DOI:** 10.2196/69750

**Published:** 2025-08-27

**Authors:** Camille E Proulx, Johanne Higgins, Thomas Vaughan, Mark Hewko, Dany H Gagnon

**Affiliations:** 1 School of Rehabilitation, Faculty of Medicine, Université de Montréal, 7077 Avenue du Parc, Montréal, QC, H3N 1X7, Canada, 1 514 343 6111; 2 Center for Interdisciplinary Research in Rehabilitation of Greater Montreal, Institut Universitaire sur la Réadaptation en Déficience Physique de Montréal, CIUSSS Centre-Sud-de-l’Île-de-Montréal, Montréal, QC, Canada; 3 Simulation and Digital Health, Medical Devices Research Centre, National Research Council Canada, Boucherville, QC, Canada; 4 Simulation and Digital Health, Medical Devices Research Centre, National Research Council Canada, Winnipeg, QC, Canada

**Keywords:** exercise, exoskeleton device, hand recovery, rehabilitation, robotic glove, stroke, virtual reality

## Abstract

**Background:**

Optimizing rehabilitation intensity using a robotic-assisted hand rehabilitation exercise (RAHRE) program coupled with a virtual environment is a promising intervention as it aligns with key neuroplasticity principles.

**Objective:**

The aim of the study is to assess the feasibility, safety, preliminary effects, and satisfaction of the 2-week RAHRE program offered as an adjunct to conventional rehabilitation.

**Methods:**

In total, 11 adults with hand hemiparesis following a recent stroke and undergoing intensive functional rehabilitation were randomized into experimental and control groups. Both groups received conventional rehabilitation therapy over a 2-week period. The experimental group received 10 additional 30-minute sessions of the RAHRE program (5 times per week), incorporating 4 hand opening and closing exercises with personalized glove assistance or resistance levels with virtual reality over the same period. Measures of feasibility (ie, attendance rate, compliance rate, repetitions per session, active training time, therapist verbal cueing, and support required), safety (ie, discomfort and adverse effects), and satisfaction (ie, satisfaction questionnaire) were collected. Functional outcomes (ie, Action Research Arm Test [ARAT], Fugl-Meyer Assessment for the Upper Extremity [FMA-UE], Box and Block Test, ABILHAND) were also assessed before and after the intervention in both groups.

**Results:**

Attendance and compliance rates in the experimental group reached 96% (48 completed training sessions of 50 planned sessions) and 95% (1432 completed training minutes of 1500 planned minutes), respectively. Participants performed a median of 2543 (IQR 2368-2951) additional movement repetitions during the RAHRE program (median repetitions per session 260, IQR 173-365; median active training time 24 minutes 39 seconds, IQR 22 minutes 26 seconds-25 minutes 51 seconds). Minimal therapist verbal cueing and support were necessary for technology use (median glove donning time 46, IQR 27-60 seconds; median independence achieved in 6, IQR 4-7 sessions). No abnormal discomfort or adverse effects were reported. Both groups showed functional improvements in ARAT, FMA-UE, Box and Block Test, and ABILHAND. For the primary outcomes (ie, ARAT and FMA-UE), the median score changes were, respectively, 4.50 (IQR 0-9) and 4.00 (IQR 3-4) in the control group, and 4.00 (IQR 1-7.5) and 5.00 (IQR 5-6) in the experimental group. Excellent overall program satisfaction (median 5/5, IQR 5-5) was reported for the RAHRE program.

**Conclusions:**

The RAHRE program, as an adjunct to conventional rehabilitation therapy, emerges as being feasible, safe, beneficial, and satisfying for adults with hand hemiparesis following a recent stroke. However, careful interpretation of the results remains recommended given the strength of the evidence. Future studies providing higher-quality evidence are needed.

## Introduction

Despite intensive functional rehabilitation efforts, 75% of people who sustained a stroke continue to experience difficulties with hand sensorimotor impairments beyond 3 months after stroke, which negatively affect their participation in daily activities [1]. To enhance poststroke recovery, evidence suggests combining various treatment modalities (eg, constraint-induced therapy, mirror therapy, robotics, and virtual reality) that integrate principles of neuroplasticity into a rehabilitation intervention [2,3]. The principles of neuroplasticity emphasize the benefits of high-intensity activity-based therapy soon after stroke [4]. However, clinical settings face challenges in achieving these high-intensity goals due to administrative constraints such as high caseloads and limited therapist availability [5]. Emerging technologies, notably robotic gloves, offer potential for integrating diverse treatment modalities into a single intervention, thereby both intensifying and enhancing rehabilitation opportunities while simultaneously alleviating any clinical or administrative burdens.

Over the past 50 years, robotic gloves have emerged as valuable assets in both clinical and research settings for promoting upper extremity function and recovery, particularly when provided for the duration of at least 30 minutes daily over a minimum period of 2 weeks [6]. These gloves can assist movement and provide haptic feedback with realistic proprioception and tactile sensations, which may improve dexterity and fine motor skills. Combining the use of a robotic glove with virtual reality represents a multimodal approach that enhances various forms of sensory feedback [7]. Beyond the rehabilitation benefits of the gloves themselves, augmented visual and auditory feedback can be provided via realistic and appealing interactive virtual reality environments created for specific motor training tasks, thereby boosting the engagement and motivation of individuals with stroke in pursuing their neurorehabilitation [8].

Advancements in glove technology have progressed, with some now available commercially, offering features tailored for rehabilitation settings such as movement tracking, kinesthetic and tactile feedback, and compatibility with virtual reality environments [9]. Recognizing the potential of these features, the Dexmo glove, a commercialized robotic glove (DextaRobotics), was selected to be coupled with our newly developed virtual environment software platform, btrained (version 2.0), specifically designed for hand rehabilitation after a stroke [10]. This coupling is now ready for clinical testing in the form of a robotic-assisted hand rehabilitation exercise (RAHRE) program to complement and intensify conventional hand rehabilitation and is the focus of this feasibility study.

This study aims to assess the feasibility, safety, and preliminary effects on hand-related functional abilities while also assessing satisfaction of a novel 2-week RAHRE program offered as an adjunct to conventional rehabilitation. Recruitment, attendance and dropout rates, learnability, and the progression of dosage over the course of the program were measured to determine the feasibility; presence of participant-specific undesirable effects such as discomfort, pain, spasticity, and skin and soft tissue integrity or the occurrence of any other adverse effects were measured to determine safety; and functional outcomes were measured to determine the effects of the program. Moreover, a questionnaire of participants’ satisfaction toward the program was completed to determine satisfaction. The study hypothesis was that the RAHRE program is feasible and can safely intensify conventional hand therapy while inducing beneficial functional changes and satisfaction for participants. It is anticipated that the findings will enrich and inform a future larger-scale efficacy study.

## Methods

### Study Design

A prospective intervention feasibility study with pre- and postevaluations was carried out over a 6-month period to assess the 2-week RAHRE program. As this was a feasibility study, no a priori power analysis was performed to determine the sample size. Participants were randomly allocated to an experimental group (RAHRE program) or a control group (conventional rehabilitation therapy) through a block randomization method. This process was facilitated by a computer-generated algorithm to ensure unbiased allocation. The randomization was centrally managed by a single designated research team member (DHG) who was not involved in the assessment or in the intervention. The use of block randomization improved the chance of maintaining balanced group sizes and minimized selection bias throughout the study. A preintervention evaluation was completed prior to group allocation, and a postintervention evaluation was completed within 40 hours upon completion of the RAHRE program. The initial evaluation included a familiarization period for all participants to acquaint themselves with the technology, ensuring that no additional exclusion criteria could hinder its use if assigned to the experimental group.

### Participants Recruitment

A nonprobabilistic consecutive sample of 11 adults who sustained a stroke and were undergoing an inpatient intensive functional neurorehabilitation program offered by a publicly funded rehabilitation center was recruited. Participants had to meet the following eligibility criteria: have hand sensorimotor impairments and functional disabilities, as determined using the subscale of the hand subsection of the Fugl-Meyer Assessment for the Upper Extremity (FMA-UE; FMA-Hand<14). Exclusion criteria included a lack of minimal motor recovery using the earlier-mentioned subscale (Dexmo glove requirement of FMA-Hand ≥1) or the inability to provide consent, to communicate in French, English, or Spanish, or to understand simple commands. All patients admitted to the rehabilitation center between September 15, 2023, and March 15, 2024, and meeting inclusion criteria were identified by a clinical research coordinator (Frédéric Messier) who communicated with their assigned occupational therapists for screening. If the patient was deemed eligible, a research professional explained the research project, verified interest in participating, and invited the person to sign the consent form.

### Ethical Considerations

The project was approved by the Rehabilitation and Physical Disability Research Ethics Committee of the Centre Intégré Universitaire de Santé et de Services Sociaux Centre-Sud-de-l’Île-de-Montréal (2023‐1822). All participants provided written informed consent prior to participation. Participant data were anonymized to ensure privacy and confidentiality. Participants received approximately US $14.56 per evaluation visit (2 visits in total) as compensation for their time and any inconvenience, as outlined in the consent form.

### Soft Robotic Glove and Software

**Figure 1.**
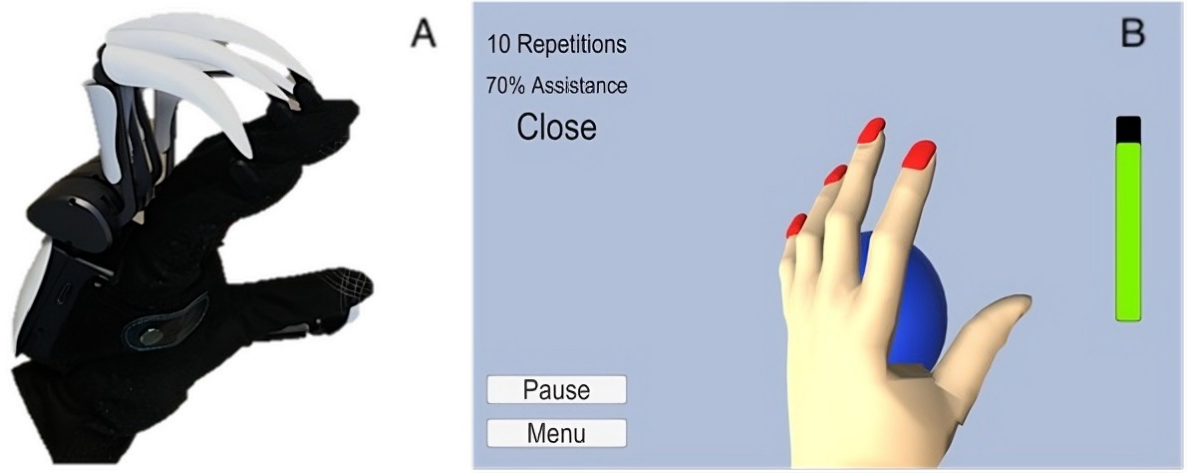
The robotic glove and software. (A) Dexmo glove. (B) Exercise level 1 in the virtual environment on btrained (version 2.0) software.

Although defined hereunder as a robotic glove, the Dexmo can be classified as a wearable hand exoskeleton or exoglove. The Dexmo enables 11 degrees of freedom of hand motion (flexion or extension and abduction or adduction of all 5 fingers and additional rotation for the thumb). It is worn on the dorsal side of the hand, and each finger is connected to the main controller at the end effector using a cloth glove (Figure 1A). The Dexmo includes a sensory module for detecting finger movements and an actuation module for adjusting force transmission to assist with movement execution [11]. The glove is linked to a virtual environment software, btrained (version 2.0), that reproduces the hand and its movements in real time through an avatar using 3D graphics.

The btrained (version 2.0) software, developed in partnership with Canada’s National Research Council using Unity software (Unity Technologies), evolved from previous iterations to address the needs of people who sustained a stroke [10]. A session with btrained (version 2.0) begins with a 1-time glove calibration for avatar movement mirroring. The session continues by having participants explore the 4 developed hand exercises associated with a spherical grip. The first level involves hand opening and closing exercises over a static 45-mm diameter virtual ball (Figure 1B). Throughout this level, glove assistance decreases by 5% for every 3 repetitions of full-finger movement, starting from 100% assistance (equivalent to ∼1.8 N) and ending with 0% assistance, then increasing by 5% in resistance up to 100% resistance (equivalent to ∼7 N). Exercise level 2 consists of a 10-minute hand opening and closing exercise with constant assistance or resistance. Exercise level 3 involves the same hand opening and closing exercise but requires the participant to follow a constant tempo (0.33 Hz) set by a metronome for the duration of 10 minutes. Exercise level 4 is similar to exercise level 3 but with a metronome tempo that increases with the number of repetitions. Visual and congruent auditory feedback signals to the participant whether or not the movement is synchronized with the metronome tempo.

At the start of each session, participants only have access to exercise level 1. Completing this level allows the software to automatically personalize the difficulty threshold for subsequent exercises (ie, levels 2, 3, and 4) to be 10% easier than level 1. Once level 1 is completed, participants have full access to the other exercises for the remainder of the 30-minute session, allowing them the freedom to choose the order and duration of each exercise. At any point during any exercise, participants can pause or end the exercise using the “Pause” and “Menu” icons on the touch screen. If participants fail to fully open or close their hand for more than 30 seconds, the exercise stops automatically. The software also tracks and displays the number of full repetitions achieved for each exercise by assessing whether the second to fifth fingers transitioned from <20° to >50° and back to <20° range of motion (ROM) of the metacarpophalangeal joints. A performance summary is also available after each exercise. This summary included the exercise duration, total number of repetitions, and level of assistance or resistance, as well as performance outcomes from previous sessions for comparison.

### Intervention: Conventional Rehabilitation Therapy and the RAHRE Program

The study aimed to demonstrate the feasibility and added value of integrating the RAHRE program into current conventional therapy (ie, pragmatic approach). In this context, the control group received only conventional therapy, and no alternative intervention was added in the context of the present feasibility study. Thus, regardless of the allocation group, all participants received conventional rehabilitation therapy (eg, massage, passive or active ROM, sensory stimulation, strengthening exercises, and functional activities of daily living) offered by their appointed rehabilitation professionals throughout the duration of the study. Overall, each participant received approximately 7.5 hours per week of individual occupational and physical therapy [12]. Participants allocated to the experimental group (ie, RAHRE program) received an additional 30-minute session on weekdays over a 2-week period (5 sessions per week for a total of 10 sessions). Each session took place at the rehabilitation center where participants underwent their regular inpatient intensive functional rehabilitation and was supervised by a registered occupational therapist (CEP) who provided support as needed to the participant. During each session, participants engaged in the different exercises available on btrained (version 2.0).

### Outcome Measures

#### Sociodemographic and Clinical Characteristics

Sociodemographic and clinical characteristics including age, sex, time since stroke onset, stroke type, most affected side, handedness, and technological experience were collected during the initial evaluation. With permission from MoCA Test Inc, a certified research member (CEP) administered the Montreal Cognitive Assessment (MoCA) to gather participants’ cognitive scores, while the Modified Ashworth Scale (MAS) was used to assess spasticity at the elbow, wrist, fingers, and thumb of the most affected arm [13,14]. Although the study did not aim to improve these parameters, these assessments were conducted to inform the development of future inclusion and exclusion criteria for the use of the Dexmo and btrained (version 2.0) software. Additionally, the FMA-Hand score for each participant was collected to confirm eligibility as previously described.

#### Feasibility

The feasibility of this intervention is structured around 3 themes: recruitment, familiarization period, and intervention. For recruitment, the number of patients admitted to the rehabilitation center, the number of those identified as potentially eligible, the number of those enrolling in the study, and the number of dropouts were collected over the course of the study. For the familiarization period, the number of participants able to don the glove independently (ie, to put on the glove and wear it on their most affected hand without the need of verbal cues nor physical support from a third party) and carry out exercise level 1 was collected. For the intervention, attendance and compliance, including the session duration of the therapy session, were collected. The therapy dose and learnability, through the number of repetitions of full movement of fingers flexion and extension per session, the active training time per session, the time for the participant to independently don the glove, the level of glove assistance or resistance, and the description of any therapist verbal cueing and support necessary to help the participants navigate through btrained (version 2.0), were also collected.

#### Safety

At the start, during, and at the end of each session, participants were asked to inform the researcher of the presence of serious adverse effects or any discomfort believed to be associated with the training sessions and specified its intensity (mild, moderate, or high). In addition, the level of hand pain was collected using a visual analog scale (VAS) at the start and end of each session.

#### Function

Five functional upper extremity assessments encompassing 2 domains of the International Classification of Functioning, Disability and Health were used to measure body function and activity and participation. Body function was assessed via the primary outcome measure, the FMA-UE, as well as grip strength and lateral pinch strength. Activity and participation were assessed using the primary outcome measure, the Action Research Arm Test (ARAT), along with the Box and Block Test (BBT) and the ABILHAND questionnaire. These assessment tools are thoroughly detailed in other sources, including the Shirley Ryan AbilityLab [15] and are known to demonstrate excellent psychometric properties in stroke populations [16-19].

#### Satisfaction

Participants in the experimental group completed a project-specific satisfaction questionnaire at the end of the RAHRE program. The questionnaire incorporated elements of both the User Satisfaction Evaluation Questionnaire [20] and the Suitability Evaluation Questionnaire (SEQ) [21] for a total of 25 questions organized around 7 sections, each including 1 to 5 questions (see Multimedia Appendix 1 to access the full version of the questionnaire). Questions incorporated into sections 1 to 6 were scored on a 5-point Likert scale ranging from 1=strongly disagree to 5=strongly agree, whereas questions incorporated in section 7 were answered on a 3-point scale depending on the item. For questions regarding duration and frequency (items 7.1-7.3), response options were: 1=adequate, 2=too short or not enough, and 3=too long or too much. For perceived effort (items 7.4-7.5), responses were: 1=mild, 2=moderate, and 3=high.

### Data Analysis

All sociodemographic and clinical characteristics, feasibility, as well as participant-specific safety measures and satisfaction data are reported with descriptive statistics (ie, median and group-median %). Functional outcome measures are analyzed by comparing individual changes in scores from pre- to postintervention. Median scores for the entire group are then extracted and interpreted against established benchmarks, such as the minimal detectable change (MDC), smallest real difference (SRD), and minimal clinically important difference (MCID), when available for the specific outcome and comparable population [22]. Changes exceeding the MDC, SRD, or MCID are deemed to indicate a significant effect in that particular functional outcome and thereafter, based on the directionality of this change, judged to be beneficial or detrimental. The proportion of participants with a change exceeding the MDC, SRD, and MCID for each outcome is also reported.

## Results

### Feasibility

#### Recruitment Process

**Figure 2.**
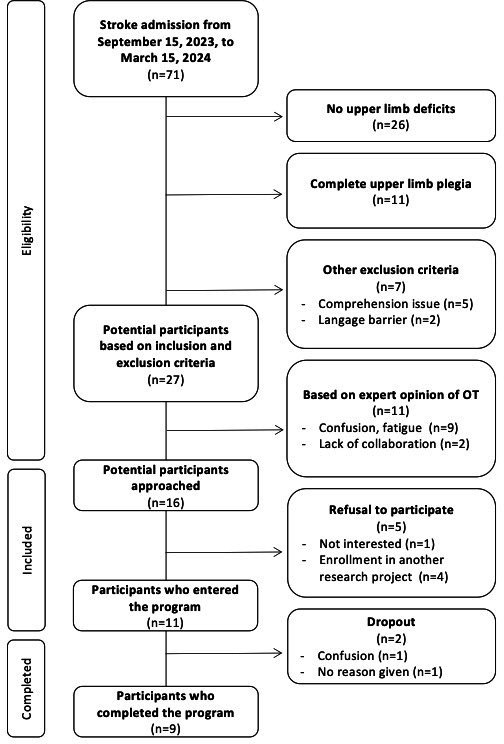
Recruitment flowchart.

Figure 2 illustrates the recruitment process and details reasons for excluding potential participants at each step. Of 71 individuals admitted to the stroke unit of the rehabilitation center between September 15, 2023, and March 15, 2024, a total of 11 enrolled in the study, resulting in a recruitment rate of 16% and a recruitment ratio of 1.83 participants per month.

#### Familiarization Period

During the familiarization period prior to initiating the intervention, most participants (8/11, 73%; except C1, C4, and E6) successfully donned the glove independently. All participants were able to complete exercise level 1. Participants were then randomized into the control (n=5) or the experimental group (n=6). Following the randomization, 1 participant from each group withdrew, resulting in a dropout rate of 18% (2/11); the participant who withdrew from the control group was discharged before the final evaluation appointment and requested data destruction, whereas the participant who withdrew from the experimental group cited being overwhelmed by learning to use technologies such as the Dexmo and btrained (version 2.0) due to age (84 years), limited technological experience, and cognitive issues (MoCA=18/30). Thus, a total of 9 participants (control group: n=4 and experimental group: n=5) completed the study. Participants’ sociodemographic and clinical characteristics are provided in Table 1. Among all participants, the highest MAS score observed was 3 for elbow spasticity, while scores for wrist and finger spasticity ranged between 0 and 2.

**Table 1.** Participants’ sociodemographic and clinical characteristics.

Participant	Age (year)	Sex	Stroke onset (months)	Stroke type	Affected side	Handedness	Technological experience^a^	MoCA^b^ (out of 30)	FMA^c^-Hand (out of 14)
Control group									
C1	52	Male	4.1	Hemorrhagic	Right	Right	B	22	3
C3	65	Female	0.83	Ischemic	Right	Right	D	15	7
C4	30	Male	1.47	Hemorrhagic	Right	Right	C	23	5
C5	80	Male	1.19	Ischemic	Left	Right	D	20	11
Experimental group									
E1	63	Male	1.87	Hemorrhagic	Left	Right	B	27	13
E2	44	Female	1.23	Ischemic	Right	Right	B	25	9
E3	74	Female	1.13	Ischemic	Left	Right	C	24	13
E5	56	Female	0.71	Hemorrhagic	Right	Right	C	15	12
E6	22	Female	1.52	Hemorrhagic	Left	Right	A	23	1

a A: expert, B: competent, C: beginner, D: ignorant.

b MoCA: Montreal Cognitive Assessment.

c FMA: Fugl-Meyer Assessment.

#### Intervention

##### Attendance and Compliance

Most participants in the experimental group (3/5) attended all training sessions, while 2 missed 1 session each, respectively, due to a technology malfunction and a scheduling conflict. Hence, the attendance rate was 96% (48 completed training sessions of 50 planned sessions). The expected session duration was 30 minutes, including the glove donning time. Most participants (3/5) completed all 30-minute sessions as planned, though 2 sessions ended earlier than expected (ie, 19 minutes 20 seconds and 28 minutes 30 seconds) due to fatigue. The overall compliance rate was 95% (1432 completed training minutes of 1500 planned minutes).

##### Therapy Dose and Learnability

**Figure 3.**
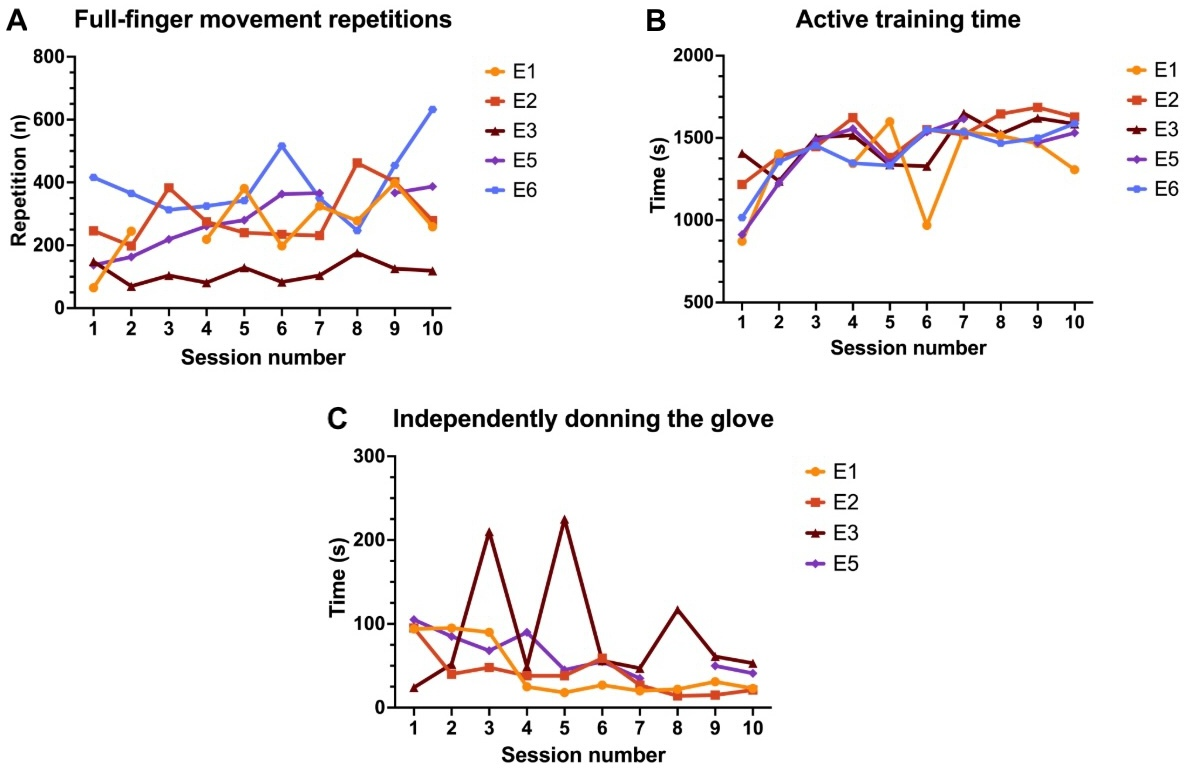
Participants’ progression in the robotic-assisted hand rehabilitation exercise (RAHRE) program. (A) Number of full-finger movement of flexion and extension repetitions (n) completed by each participant in each session. (B) Active training time (seconds) for each participant in each session. (C) Time required (seconds) to independently don the glove for each participant capable of independent donning, for each session.

A summary of the number of repetitions of full-finger movement of flexion and extension, their active training time, and the time for participants to independently don the glove over the course of the study is reported in Figure 3. The number of full-movement finger flexion and extension repetitions per session ranged from 65 to 632 (median 260, IQR 173-365 repetitions), with a median active training time of 24 minutes 39 seconds (IQR 22 minutes 26 seconds-25 minutes 51 seconds). This resulted in a median intensity ratio of 10.2 (IQR 6.6-13.1) repetitions per minute of active training and 8.2 (IQR 4.4-10.5) repetitions per minute of total training session. Independent donning times ranged from 14 to 225 seconds (median 46, IQR 27-60 seconds). Only 1 participant (E6) required continuous therapist support to don the glove until the end of the program due to severe hand impairments. The median donning time for this participant with therapist support was 181 (IQR 162-195) seconds. Participants predominantly used the Dexmo glove with resistance (n=4) rather than assistance (n=1). Therapist verbal cueing and support required to navigate btrained (version 2.0) was only needed during the first 7 sessions, with a median of 6 (IQR 4-7) sessions to reach full independence. By the fifth session, 3 of 5 participants were completely autonomous. Independence was more difficult to achieve for the remaining 2 (E3 and E5), who required support, respectively, in 70% (7/10) and 40% (4/10) of sessions, while the other 3 needed assistance in no more than 20% (2/10) of sessions.

### Safety

No serious adverse effect associated with the RAHRE program was reported. Most participants (4/5) experienced mild to moderate muscle fatigue in the forearm during or at the end of at least 1 session. Some participants took short breaks due to muscle fatigue during a session, and others deemed it unnecessary. Muscle fatigue never leads to a session termination. No instances of increased spasticity, hypertonicity, or other discomforts such as skin lesions, stiffness, confusion, or dizziness were observed or reported by participants during or after any of the sessions.

Hand pain was reported by 1 participant (E5) before every training session (median VAS score 2.1 per 10 cm, IQR 1.7-2.9 cm), with an increase in pain reported at the end of each session never exceeding 1 cm (median increase on VAS 0.1 per 10 cm, IQR −0.2 to 0.4 cm).

### Preliminary Functional Effects

Tables2  3 present the individual pre- and postintervention scores and overall change in scores for each participant, along with the group median. For each functional outcome, the tables also display the corresponding MDC or SRD, as well as the MCID, when available. Both groups demonstrated functional improvements on the ARAT, FMA-UE, and BBT, with median score changes exceeding the respective MDC or SRD values. In the control group, 50% of participants exceeded the MDC for both the ARAT and FMA-UE, and 75% exceeded the SRD for the BBT. In the experimental group, 60% of participants exceeded the MDC for the ARAT, 80% for the FMA-UE, and 60% exceeded the SRD for the BBT. For the ABILHAND, both groups also showed improvements, with median score differences exceeding the MCID. In the control group, 75% of participants exceeded the MCID. In the experimental group, 80% exceeded the MCID, while 20% showed changes below the MCID threshold in the negative direction.

**Table 2.** Individual pre- and postintervention scores, change scores, and group data scores for Action Research Arm Test (ARAT), Fugl-Meyer Assessment for the Upper Extremity (FMA-UE), and grip strength.

Group	ARAT (points out of 57) (MDC^a^=3.5 points; MCID^b^=12‐17 points [23,24])			FMA-UE (points out of 66) (MDC=3.5 points; MCID=9‐10 points [24,25])			Grip strength (kg) (SRD^c^=2.9 kg; MCID=5‐6.2 kg [16,23])		
	Prescore	Postscore	Δ^d^	Prescore	Postscore	Δ	Prescore	Postscore	Δ
Control group									
Individual data									
C1	0.0	0.0	0.0	10.0	11.0	1.0	7.0	6.0	−1.0
C3	28.0	37.0	9.0	50.0	57.0	7.0	11.3	12.3	1.0
C4	1.0	10.0	9.0	17.0	26.0	9.0	0.0	4.7	4.7
C5	54.0	54.0	0.0	53.0	54.0	1.0	30.0	30.8	0.8
Group data									
Median (IQR)	14.5 (0.8-34.5)	23.5 (7.5-41.3)	4.5^e^ (0.0-9.0)	33.5 (15.3-50.8)	40.0 (22.3-54.8)	4.0^e^ (1.0-7.5)	9.2 (5.3-16.0)	9.2 (5.7-17.0)	0.9 (0.4-1.9)
Experimental group									
Individual data									
E1	54.0	57.0	3.0	49.0	57.0	8.0	25.0	26.0	1.0
E2	39.0	55.0	16.0	49.0	54.0	5.0	8.3	9.3	1.0
E3	55.0	57.0	2.0	57.0	59.0	2.0	12.0	16.3	4.3
E5	51.0	55.0	4.0	54.0	60.0	6.0	20.7	22.7	2.0
E6	0.0	4.0	4.0	13.0	18.0	5.0	4.0	6.7	2.7
Group data									
Median (IQR)	51.0 (39.0-54.0)	55.0 (55.0-57.0)	4.0^e^ (3.0-4.0)	49.0 (49.0-54.0)	57.0 (54.0-59.0)	5.0^e^ (5.0-6.0)	12.0 (8.3-20.7)	16.3 (9.3-22.7)	2.0 (1.0-2.7)

a MDC: minimal detectable change.

b MCID: minimal clinically important difference.

c SRD: smallest real difference.

d Change in score between pre- and postscores.

e Median score changes exceeding the respective MDC, SRD, or MCID values.

**Table 3.** Individual pre- and postintervention scores, change scores, and group data scores for lateral pinch strength, Box and Block Test (BBT), and ABILHAND.

Group	Lateral pinch strength (kg) (SRD^a^=1.4 kg [16])			BBT (blocks per minute) (SRD=5.5 blocks per minute [16])			ABILHAND (per logit) (MCID^b^=0.26‐0.35 logits [19])		
	Prescore	Postscore	Δ^c^	Prescore	Postscore	Δ	Prescore	Postscore	Δ
Control group									
Individual data									
C1	2.2	2.2	0.0	0.0	2.0	2.0	0.0	0.3	0.3
C3	2.8	2.8	0.0	20.0	26.0	6.0	1.1	2.2	1.1
C4	0.3	1.2	0.9	0.0	7.0	7.0	−1.0	−0.4	0.7
C5	7.7	8.0	0.3	33.0	40.0	7.0	3.8	4.3	0.5
Group data									
Median (IQR)	2.5 (1.7- 4.0)	2.5 (1.9-4.1)	0.2 (0.0-0.5)	10.0 (0.0-23.3)	16.5 (5.8-29.5)	6.5^d^ (5.0-7.0)	0.5 (−0.3-1.8)	1.2 (0.1-2.7)	0.6^d^ (0.5-2.4)
Experimental group									
Individual data									
E1	5.8	7.0	1.3	27.0	30.0	3.0	2.6	4.3	1.7
E2	2.0	2.1	0.1	27.0	36.0	9.0	−0.8	2.0	2.8
E3	4.5	5.6	1.1	15.0	23.0	8.0	1.8	2.4	0.6
E5	4.2	6.5	2.3	35.0	42.0	7.0	−1.3	1.1	2.4
E6	0.5	0.7	0.2	0.0	2.0	2.0	0.8	0.1	−0.6
Group data									
Median (IQR)	4.2 (2.0-4.5)	5.6 (2.1-6.5)	1.1 (0.2-1.3)	27.0 (15.0-27.0)	30.0 (23.0-36.0)	7.0^d^ (3.0-8.0)	0.8 (−0.8-1.8)	2.4 (1.1-2.4)	1.7^d^ (0.6-2.4)

a SRD: smallest real difference.

b MCID: minimal clinically important difference.

c Change in score between pre- and postscores.

d Median score changes exceeding the respective minimal detectable change, SRD, or MCID values.

### Satisfaction

Participants’ median satisfaction scores for each section of the questionnaire are reported in Tables4  5. Overall, participants expressed high satisfaction with the RAHRE program, Dexmo glove, and btrained (version 2.0). The program was found to be both satisfying and motivating, with participants acknowledging its high learnability and ease of use. Additionally, participants strongly agreed with the perceived health benefits associated with the RAHRE program.

**Table 4.** Satisfaction median score per section for each participant.

Domains: part 1‐6	E1	E2	E3	E5	E6
1. Overall satisfaction with the RAHRE^a^ program	5	5	5	4.5	5
2. Satisfaction with the robotic glove	5	5	5	4	5
3. Satisfaction with the virtual environment system attributes	5	5	5	4	3.5
4. Satisfaction and motivation with exercise program	5	5	4.5	5	2
5. Learning how to use the robotic glove coupled to the virtual environment system	5	4	5	5	4
6. Perceived health benefits	5	5	5	5	3

a RAHRE: robotic-assisted hand rehabilitation exercise.

**Table 5.** Participant’s level of satisfaction with the setting of the program.

Domains: part 7: satisfaction with the setting of the RAHRE^a^ program	E1	E2	E3	E5	E6
7.1. The total duration of the program, which took place over a period of 2 weeks, was	Adequate	Adequate	Adequate	Adequate	Adequate
7.2. The number of exercise sessions (5 times per week) is	Adequate	Adequate	Too much	Adequate	Adequate
7.3. The duration of each exercise session, which is approximately 30 minutes, is	Adequate	Adequate	Adequate	Adequate	Adequate
7.4. I perceived a level of physical exertion _____ during exercise sessions	Mild	Moderate	Moderate	Moderate	High
7.5. I perceived a level of cognitive effort (attention, concentration, etc) ____ during exercise sessions	Moderate	Moderate	High	High	Moderate

a RAHRE: robotic-assisted hand rehabilitation exercise.

## Discussion

Although based on a small sample, this preliminary feasibility study suggests that adding a 30-minute high-intensity, hand-specific RAHRE program to conventional rehabilitation is feasible and safe and holds promise for improving hand function and achieving high participant satisfaction.

### RAHRE Program as an Adjunct to Conventional Inpatient Rehabilitation Is Feasible

The results of this study demonstrate feasibility, first, by confirming a recruitment ratio of 1.83 participants per month, which is similar to the ratio reported in other comparable studies (ranging between 1.5 and 1.9 participants per month) [26,27]. When the project was introduced to potential participants, there were minimal refusals (n=5), indicating that the intervention was appealing and motivating for individuals with stroke. In fact, the main reason for declining participation was their interest in enrolling in another research project, deployed simultaneously, and that aligned better with their therapeutic preferences and needs. The dropout rate of 18% exceeded the median dropout rate of 6% reported in a systematic review on recruitment in stroke rehabilitation randomized controlled trials [26]. Nonetheless, the dropout rate in this study was lower when compared to another study involving a similar number of participants and interventions, which had a dropout rate of 30% [28].

Second, the attendance rate was excellent (48 completed training sessions out of 50 planned sessions, 96%), indicating that both the frequency and duration of the novel RAHRE program (5 sessions per week; 30 minutes per session) are feasible. Such an engagement was facilitated by offering the novel program during inpatient rehabilitation, thereby reducing common attendance barriers often encountered in outpatient rehabilitation, such as transportation issues and the unavailability of caregivers to accompany participants [29]. However, applying the intervention during inpatient rehabilitation comes with other issues such as scheduling challenges due to multiple interventions and the development of fatigue as the day progresses. Future trials should carefully consider participant fatigue when scheduling the RAHRE program. Independent use of the technology, without constant therapist supervision, and ensuring its availability at all times would enable more scheduling flexibility.

### RAHRE Program Can Be Performed Independently

The results of this study confirm that participants progressively achieved autonomy and independence in performing the RAHRE program. First, despite expectations of challenges in donning the Dexmo glove for individuals who have had a stroke, most participants successfully donned the glove independently, efficiently, and with ease, never exceeding the 5‐ to 10-minute donning period recommended by occupational therapists to optimize therapy time [30]. In this study, the donning process typically took less than 1 minute, with or without therapist support, and there was a noticeable trend of decreased donning time across sessions, confirming improved efficiency with practice. Based on the findings, which should be interpreted with caution due to the small sample size, it is important to note that a score ≤5 on the FMA-Hand makes it challenging for individuals to don the glove independently. Conversely, as mentioned, a MAS score of ≤2 seems to be acceptable for the use of the glove.

Second, after donning the glove, the next crucial part of the intervention was for participants to become independent in navigating through btrained (version 2.0). The results confirm that within less than 1.5 weeks, all participants had achieved independence in using btrained (version 2.0). These findings are consistent with those of a similar study involving a 4-week protocol of robot-assisted poststroke rehabilitation [31], where supervised therapy gradually transitioned to unsupervised sessions after a 2-week period.

However, achieving this level of independence was markedly more challenging for 2 participants. To explain this discrepancy, participants’ sociodemographic characteristics (cognition level via MoCA, age, and technological experience) were closely examined for potential determinants on the acquisition of independence in navigating through btrained (version 2.0). Initially, participants’ MoCA score was considered, recognizing that a decline in cognitive functions can impact the ability to manage technology [32]. A MoCA score below 24, which is close to the known cutoff of <26 for mild cognitive impairment, may necessitate more therapist verbal cueing and support [33]. However, this criterion alone may not fully explain the phenomenon. The participant with the lowest MoCA score (15/30) did not require the most therapist verbal cueing and support. Instead, the participant who required the greatest amount of cueing and support, E3 (7/10 sessions), was the oldest at 74 years of age (9 years older than the second-oldest participant) and had no cognitive impairment. This aligns with the understanding that the recall or recognition of information encoded, a process required to navigate autonomously through btrained (version 2.0), tends to decrease with age [34]. As for experience with technologies, the 2 participants requiring the most verbal cueing and support were both technology neophytes, having solely basic knowledge of the internet. Interestingly, cognition level, age, and experience with technologies were the reasons for withdrawal given by the participant who dropped out of the experimental group. Experience with a smartphone or computer could be considered as an inclusion criterion to increase independence in navigating through btrained (version 2.0) in a future clinical study.

### RAHRE Program Intensifies Hand Neurorehabilitation

The results of this study provide compelling evidence that the RAHRE program can intensify functional hand-specific movements (+260 repetitions per session for 10 days) known to be crucial to improve functional outcomes in this population. However, this intensification was inherent to the study design, where the experimental group was expected to engage in more exercise compared to the control group. Indeed, the increased exercise time for the experimental group was a direct result of the research protocol, which anticipated higher intensity of rehabilitation due to the greater amount of exercise performed. However, what is particularly noteworthy is the rapid attainment of a high level of independence by participants. The user-friendly design of the robotic glove, combined with the virtual environment, facilitated this rapid proficiency with the equipment. This design not only supports the feasibility of the RAHRE program with minimal therapist supervision but also allows participants to engage in rehabilitation activities outside conventional therapy hours and without overburdening therapists. This autonomy in exercise execution is a crucial aspect of intensifying rehabilitation, as it allows for greater flexibility and potentially enhances overall therapeutic effectiveness. The results of this study are in line with previous studies on the feasibility of unsupervised robot-assisted therapy using an actuated upper extremity device in a clinical setting that resulted in a significant increase in therapy dose [31]. Both this study and others are based on strategies aimed at enhancing practice opportunities and fostering active engagement in rehabilitation [35] by providing, beyond conventional rehabilitation hours, a novel therapy avenue for increasing therapy doses with minimal therapist supervision within clinical settings. The high portability of the Dexmo and its virtual environment makes it possible for the RAHRE program to be extended to home-based rehabilitation.

In addition to enhancing therapy opportunities for rehabilitation, therapy dose, including the number of repetitions, resistance levels during each repetition, and the time dedicated to exercising, remains one of the key strategies to intensify hand neurorehabilitation. The median observed repetition intensity ratio of 10.2 repetitions per active training minute and 8.2 repetitions per total session minute is above what is seen in a conventional rehabilitation session. For instance, in studies focusing on the amount of repetition achieved in conventional rehabilitation sessions, the average intensity ratio was below 2.4‐3.8 repetitions per minute [36,37], while studies focusing on high-repetition of task-specific training reported an average intensity ratio between 6.1 and 7.1 repetitions per minute [38,39]. Thus, the RAHRE program offers a higher intensity ratio than conventional therapy alone and surpasses similar studies on high-intensity training. Moreover, despite some variations, participants generally increased the number of repetitions across sessions. Lower repetition counts may have been influenced by variations in the level of glove assistance or resistance provided during exercises (eg, the higher the glove resistance, the smaller the number of repetitions). Fatigue levels toward the end of the day could also negatively influence repetitions. To optimize the functional benefits of time dedicated to exercising, the second Stroke Recovery and Rehabilitation Roundtable recommends intervention doses of more than 1 hour per day on a task [2]. While each participant received approximately 7.5 hours per week of individual conventional occupational and physiotherapy [12], not all of these hours were related to task practice. In fact, only one-third of therapy time is dedicated to task practice [40]. Therefore, supplementing conventional therapy with the 30-minute RAHRE program could help bridge this gap and align more closely with Stroke Recovery and Rehabilitation Roundtable recommendations. It remains uncertain how this intensification translates to increased effect and efficacy, and studies using a higher level of evidence (ie, randomized controlled trial) are needed.

### RAHRE Program Induces Beneficial Effects

The results of this study do not provide conclusive evidence that the RAHRE program induces significant beneficial effects in terms of functional capacity, particularly for grasp-related activities. While median changes in scores for both primary outcomes, ARAT and FMA-UE, were greater than the MDC or SRD, indicating a true change, they did not exceed the MCID. This modest yet encouraging impact may be partially attributed to the brief period of the intervention (eg, duration), combined with its early implementation in the poststroke period, a phase during which spontaneous recovery is frequently observed [41]. Given the neuroplastic changes and endogenous repair mechanisms active during this acute phase, the functional improvements observed in both groups likely reflect, at least in part, natural recovery processes rather than the effects of the intervention alone. To more accurately isolate and assess intervention-specific effects, longer intervention periods, typically exceeding 5 weeks, are generally more effective in eliciting measurable gains in fine motor control.

Moreover, the small sample size of our study limited the ability to perform comparative analyses, such as *t* tests, and to detect any potential superior effects of the RAHRE program over conventional therapy alone. Nevertheless, previous meta-analyses have confirmed that rehabilitation with robotic gloves results in significant functional improvements compared to conventional rehabilitation alone [6]. Future efficacy studies with larger sample sizes are needed to assess the potential superiority of the RAHRE program compared to conventional therapy. Based on a power analysis using G*Power (version 3.1.9.4; Heinrich Heine University Düsseldorf) software, such studies should aim for a sample size of 106 participants (53 per group) to achieve a statistical power of 0.8 with a 2-tailed *t* test at a significance level of .05. This calculation accounts for a medium-large effect size (Cohen *d*=0.6), normal distribution of outcomes, and an 18% dropout rate observed in this study. In these future studies, the control group should receive an alternative hand-targeted exercise therapy of equal duration and intensity as the experimental group, but without the glove and virtual reality. This would allow for a more accurate assessment of the superiority of the RAHRE program compared to nontechnological alternatives.

The selection of outcome measures to capture changes both in gross and fine hand sensorimotor recovery and their impacts on functional activities was a challenge in this study. Typically, gross motor movements are inherently easier to perform compared to tasks requiring fine dexterity [42]. Given that the RAHRE program led to beneficial changes in both overall upper extremity outcomes (FMA-UE and ARAT) and gross dexterity outcome (BBT), it would be relevant to further explore the effect of the RAHRE program on fine dexterity. To do so, incorporating the Nine-Hole Peg Test as an additional outcome would be effective to measure functional progress in terms of fine dexterity, especially for individuals with less severe impairments [43].

### RAHRE Program Is Safe and Satisfying

The results of this study support that the RAHRE program is clinically safe and satisfying for participants. While it is not uncommon for studies involving virtual reality to report instances of dizziness, soreness, headaches, nausea, or visual disturbance [44], none were observed in this study. In fact, no serious adverse effect was documented. Participants only reported mild to moderate muscular fatigue and physical exertion aligning with the American Heart Association and American Stroke Association for exercise intensity recommendations to prevent subsequent stroke and other cardiovascular events [45]. As for the presence of moderate to high cognitive effort, the results are consistent with studies showing positive effects of motor-cognitive interventions on physical and cognitive functioning [46,47]. These findings, along with the high level of satisfaction, are similar in most part to those of Warland et al [48], who observed unaccustomed muscular pain, cognitive fatigue, perceived improvements in impairments and functional use, and a high level of motivation among participants using a virtual reality–based upper-extremity stroke rehabilitation device. Nonetheless, before the RAHRE program is adopted as a routine intervention in clinical practice, it would be relevant to evaluate its safety in a broader framework, including adherence to standard protocols for safety evaluation, testing, and risk management for medical devices.

### Limitations

Some limitations should be acknowledged to contextualize the results of this feasibility study. First, the small sample size and the considerable variability in participant characteristics (eg, age, sex, stroke type, and affected side) and baseline scores within each group need to be highlighted. It did limit the statistical power to detect between-group differences and identify statistically or clinically meaningful changes attributable to the intervention. However, it is crucial to emphasize that the primary aim of this feasibility study was to assess the practical implementation of the intervention rather than to establish its efficacy. As such, while the study provides insights into feasibility, it underscores the need for future research with larger sample sizes and more rigorous research designs to strengthen evidence regarding the efficacy of the RAHRE program. A larger sample size would also enable subgroup analyses based on various sociodemographic and clinical factors (eg, age, sex, stroke type, most affected side, and FMA-Hand score), providing a more nuanced understanding of how these variables may influence outcomes. Second, the high level of satisfaction toward the program may reflect a desirability bias, as the same person (CEP) both supervised the intervention and collected satisfaction data. To mitigate this bias in future studies, an independent evaluator should conduct the satisfaction assessments. Third, blinding to randomization was maintained only during the initial evaluation, as the occupational therapist (CEP) was also administering the intervention. Participant blinding was not feasible due to the intervention’s nature. Fourth, the number of repetitions performed may have been underestimated. In fact, the number of movement repetition compatibilized in btrained (version 2.0) only accounted for full flexion and extension movements. Similarly, for exercises 3 and 4, only repetitions performed in sync with the metronome were counted, excluding any full flexion and extension movements that deviated from the tempo provided. Finally, achieving ROM targets in extension poses challenges compared to flexion due to longer-lever arms when participants’ fingers were in an extension motion. To better accommodate these biomechanical differences, adjusting glove resistance torque levels independently for flexion and extension would be crucial in future iterations of the RAHRE program.

### Conclusions

The RAHRE program emerges as a feasible intervention from a clinical perspective that demonstrates encouraging beneficial effects for hand functional recovery. Moreover, the intervention remains safe and satisfying for people who sustained a stroke currently undergoing inpatient intensive functional rehabilitation (ie, end users). This innovative program, which combines the Dexmo glove and the btrained (version 2.0) platform, offers an avenue to intensify hand rehabilitation. Notably, the RAHRE program can be used independently by individuals with stroke with minimal support from a rehabilitation professional. Undertaking an efficacy study on a broader scale and for an extended duration would greatly enhance the strength of currently available evidence and inform practical applications of this novel intervention in the future.

## Supplementary material

10.2196/69750 Multimedia Appendix 1 Project-specific satisfaction questionnaire.

## Abbreviations

ARAT: Action Research Arm Test
BBT: Box and Block Test
FMA-UE: Fugl-Meyer Assessment for the Upper Extremity
MAS: Modified Ashworth Scale
MCID: minimal clinically important difference
MDC: minimal detectable change
MoCA: Montreal Cognitive Assessment
RAHRE: robotic-assisted hand rehabilitation exercise
ROM: range of motion
SRD: smallest real difference
VAS: visual analog scale

## References

[1] Parker VM, Wade DT, Langton Hewer R (1986). Loss of arm function after stroke: measurement, frequency, and recovery. Int Rehabil Med.

[2] Bernhardt J, Hayward KS, Dancause N (2019). A stroke recovery trial development framework: consensus-based core recommendations from the second stroke recovery and rehabilitation roundtable. Int J Stroke.

[3] Dimyan MA, Cohen LG (2011). Neuroplasticity in the context of motor rehabilitation after stroke. Nat Rev Neurol.

[4] Teasell R, Foley N, Salter K, Bhogal S, Jutai J, Speechley M (2009). Evidence-Based Review of Stroke Rehabilitation: executive summary, 12th edition. Top Stroke Rehabil.

[5] Mudge S, Hart A, Murugan S, Kersten P (2017). What influences the implementation of the New Zealand stroke guidelines for physiotherapists and occupational therapists?. Disabil Rehabil.

[6] Ko MJ, Chuang YC, Ou-Yang LJ, Cheng YY, Tsai YL, Lee YC (2023). The application of soft robotic gloves in stroke patients: a systematic review and meta-analysis of randomized controlled trials. Brain Sci.

[7] Proulx CE, Louis Jean MT, Higgins J, Gagnon DH, Dancause N (2022). Somesthetic, visual, and auditory feedback and their interactions applied to upper limb neurorehabilitation technology: a narrative review to facilitate contextualization of knowledge. Front Rehabil Sci.

[8] Feintuch U, Raz L, Hwang J (2006). Integrating haptic-tactile feedback into a video-capture-based virtual environment for rehabilitation. Cyberpsychol Behav.

[9] Caeiro-Rodríguez M, Otero-González I, Mikic-Fonte FA, Llamas-Nistal M (2021). A systematic review of commercial smart gloves: current status and applications. Sensors (Basel).

[10] Proulx CE, Higgins J, Vincent C, Vaughan T, Hewko M, Gagnon DH (2023). User-centered development process of an operating interface to couple a robotic glove with a virtual environment to optimize hand rehabilitation following a stroke. J Rehabil Assist Technol Eng.

[11] Gu X, Zhang Y, Sun W, Bian Y, Zhou D, Kristensson PO Dexmo: an inexpensive and lightweight mechanical exoskeleton for motion capture and force feedback in VR.

[12] Richards CL, Malouin F, Nadeau S (2018). Amount and content of sensorimotor therapy delivered in three stroke rehabilitation units in Quebec, Canada. Physiother Can.

[13] Charalambous CP (2014). Classic Papers in Orthopaedics.

[14] Katz RT, Rovai GP, Brait C, Rymer WZ (1992). Objective quantification of spastic hypertonia: correlation with clinical findings. Arch Phys Med Rehabil.

[15] (2024). Rehabilitation Measures Database. Shirley Ryan AbilityLab.

[16] Chen HM, Chen CC, Hsueh IP, Huang SL, Hsieh CL (2009). Test-retest reproducibility and smallest real difference of 5 hand function tests in patients with stroke. Neurorehabil Neural Repair.

[17] Mathiowetz V, Weber K, Volland G, Kashman N (1984). Reliability and validity of grip and pinch strength evaluations. J Hand Surg Am.

[18] Platz T, Pinkowski C, van Wijck F, Kim IH, di Bella P, Johnson G (2005). Reliability and validity of arm function assessment with standardized guidelines for the Fugl-Meyer Test, Action Research Arm Test and Box and Block Test: a multicentre study. Clin Rehabil.

[19] Wang TN, Lin KC, Wu CY, Chung CY, Pei YC, Teng YK (2011). Validity, responsiveness, and clinically important difference of the ABILHAND questionnaire in patients with stroke. Arch Phys Med Rehabil.

[20] Gil-Gómez JA, Manzano-Hernández P, Albiol-Pérez S, Aula-Valero C, Gil-Gómez H, Lozano-Quilis JA (2017). USEQ: a short questionnaire for satisfaction evaluation of virtual rehabilitation systems. Sensors (Basel).

[21] José-Antonio GG, Pilar MH, Sergio AP, Carmen AV, Hermenegildo GG, José-Antonio LQ SEQ: Suitability Evaluation Questionnaire for virtual rehabilitation systems. Application in a virtual rehabilitation system for balance rehabilitation.

[22] Dvir Z (2015). Difference, significant difference and clinically meaningful difference: the meaning of change in rehabilitation. J Exerc Rehabil.

[23] Lang CE, Edwards DF, Birkenmeier RL, Dromerick AW (2008). Estimating minimal clinically important differences of upper-extremity measures early after stroke. Arch Phys Med Rehabil.

[24] Lin JH, Hsu MJ, Sheu CF (2009). Psychometric comparisons of 4 measures for assessing upper-extremity function in people with stroke. Phys Ther.

[25] Narayan Arya K, Verma R, Garg RK (2011). Estimating the minimal clinically important difference of an upper extremity recovery measure in subacute stroke patients. Top Stroke Rehabil.

[26] McGill K, Sackley CM, Godwin J, McGarry J, Brady MC (2020). A systematic review of the efficiency of recruitment to stroke rehabilitation randomised controlled trials. Trials.

[27] Norouzi-Gheidari N, Archambault PS, Monte-Silva K (2021). Feasibility and preliminary efficacy of a combined virtual reality, robotics and electrical stimulation intervention in upper extremity stroke rehabilitation. J Neuroeng Rehabil.

[28] Chen J, Nichols D, Brokaw EB, Lum PS (2017). Home-based therapy after stroke using the hand spring operated movement enhancer (HandSOME). IEEE Trans Neural Syst Rehabil Eng.

[29] Chen AWL, Koh YT, Leong SWM, Ng LWY, Lee PSY, Koh GCH (2014). Post community hospital discharge rehabilitation attendance: self-perceived barriers and participation over time. Ann Acad Med Singap.

[30] Proulx CE, Beaulac M, David M (2020). Review of the effects of soft robotic gloves for activity-based rehabilitation in individuals with reduced hand function and manual dexterity following a neurological event. J Rehabil Assist Technol Eng.

[31] Devittori G, Dinacci D, Romiti D (2024). Unsupervised robot-assisted rehabilitation after stroke: feasibility, effect on therapy dose, and user experience. J Neuroeng Rehabil.

[32] Malinowsky C, Almkvist O, Kottorp A, Nygård L (2010). Ability to manage everyday technology: a comparison of persons with dementia or mild cognitive impairment and older adults without cognitive impairment. Disabil Rehabil Assist Technol.

[33] Nasreddine ZS, Phillips NA, Bédirian V (2005). The Montreal cognitive assessment, MoCA: a brief screening tool for mild cognitive impairment. J Am Geriatr Soc.

[34] Brickman AM, Stern Y, Hof PR, Mobbs CV (2009). Handbook of the Neuroscience of Aging.

[35] Eng XW, Brauer SG, Kuys SS, Lord M, Hayward KS (2014). Factors affecting the ability of the stroke survivor to drive their own recovery outside of therapy during inpatient stroke rehabilitation. Stroke Res Treat.

[36] Lang CE, MacDonald JR, Gnip C (2007). Counting repetitions: an observational study of outpatient therapy for people with hemiparesis post-stroke. J Neurol Phys Ther.

[37] Scrivener K, Sherrington C, Schurr K, Treacy D (2011). Many participants in inpatient rehabilitation can quantify their exercise dosage accurately: an observational study. J Physiother.

[38] Baniña MC, Molad R, Solomon JM (2022). Exercise intensity of the upper limb can be enhanced using a virtual rehabilitation system. Disabil Rehabil Assist Technol.

[39] Waddell KJ, Birkenmeier RL, Moore JL, Hornby TG, Lang CE (2014). Feasibility of high-repetition, task-specific training for individuals with upper-extremity paresis. Am J Occup Ther.

[40] Ada L, Mackey F, Heard R, Adams R (1998). Stroke rehabilitation: does the therapy area provide a physical challenge?. Aust J Physiother.

[41] Cassidy JM, Cramer SC (2017). Spontaneous and therapeutic-induced mechanisms of functional recovery after stroke. Transl Stroke Res.

[42] Hijikata N, Kawakami M, Ishii R (2020). Item difficulty of Fugl-Meyer Assessment for Upper Extremity in persons with chronic stroke with moderate-to-severe upper limb impairment. Front Neurol.

[43] Jacob-Lloyd HA, Dunn OM, Brain ND, Lamb SE (2005). Effective measurement of the functional progress of stroke clients. British Journal of Occupational Therapy.

[44] Bargeri S, Scalea S, Agosta F (2023). Effectiveness and safety of virtual reality rehabilitation after stroke: an overview of systematic reviews. EClinicalMedicine.

[45] Billinger SA, Arena R, Bernhardt J (2014). Physical activity and exercise recommendations for stroke survivors: a statement for healthcare professionals from the American Heart Association/American Stroke Association. Stroke.

[46] Gheysen F, Poppe L, DeSmet A (2018). Physical activity to improve cognition in older adults: can physical activity programs enriched with cognitive challenges enhance the effects? A systematic review and meta-analysis. Int J Behav Nutr Phys Act.

[47] Pichierri G, Wolf P, Murer K, de Bruin ED (2011). Cognitive and cognitive-motor interventions affecting physical functioning: a systematic review. BMC Geriatr.

[48] Warland A, Paraskevopoulos I, Tsekleves E (2019). The feasibility, acceptability and preliminary efficacy of a low-cost, virtual-reality based, upper-limb stroke rehabilitation device: a mixed methods study. Disabil Rehabil.

