# Supplementary material for: Poststroke Neurorehabilitation Using a Soft Robotic Glove Combined With a Virtual Environment: Preliminary Study on Feasibility, Safety, Effects, and User Satisfaction
Source: JMIR Neurotechnol. 2025 Aug 27;4:e69750. doi: 10.2196/69750 (PMC12671301; doi:10.2196/69750)
Supplement: Multimedia Appendix 1 [file neuro-v4-e69750-s001.docx]

| Questions | Strongly disagree  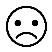 |  | |  |  | | Strongly agree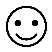 |
| --- | --- | --- | --- | --- | --- | --- | --- |
|  | 1 | 2 | | 3 | 4 | | 5 |
| **1. Overall satisfaction with the RAHRE Program** | | | | | | | |
| 1.1. I am generally satisfied with the program combining a robotic glove to a virtual environment I recently completed. |  |  | |  |  | |  |
| 1.2. I would recommend participation in the RAHRE Program to others who have had a stroke. |  |  | |  |  | |  |
| **2. Satisfaction with the robotic glove** | | | | | | | |
| 2.1. The overall appearance of the robotic glove is pleasant. |  |  | |  |  | |  |
| 2.2 It is easy to don and doff the robotic glove without the help of a therapist. |  |  | |  |  | |  |
| 2.3 Wearing the robotic glove is comfortable. |  |  | |  |  | |  |
| 2.4 The weight of the robotic glove has no effect on my use. |  |  | |  |  | |  |
| 2.6 The finger movement generated by the robotic glove is similar to a normal finger movement. |  |  | |  |  | |  |
| **3. Satisfaction with the virtual environment system attributes** | | | | | | | |
| 3.1 You felt that the virtual environment of the system was realistic. |  |  | |  |  | |  |
| 3.2 The system interface was easily usable. |  |  | |  |  | |  |
| **4. Satisfaction and motivation with the RAHRE Program** | | | | | | | |
| 4.1 The variety of exercises was adequate. |  |  | |  |  | |  |
| 4.2 The level of difficulty of the exercises was adequate. |  |  | |  |  | |  |
| 4.3 The exercise objectives were motivating. |  |  | |  |  | |  |
| 4.4 Exercises should include more of the wrist, elbow, and shoulder. |  |  | |  |  | |  |
| **5. Learning how to use the robotic glove coupled to the virtual environment system** | | | | | | | |
| 5.1 The sounds emitted by the robotic glove coupled to the virtual environment helped me to perform better during exercises. |  |  | |  |  | |  |
| 5.2 I am satisfied with the level of proficiency I have achieved with the robotic glove and the exercises in the virtual environment system at the end of the program. |  |  | |  |  | |  |
| 5.3 The instructions and feedback provided by the therapist helped my learning process to use the robotic glove and navigate through the virtual environment system. |  |  | |  |  | |  |
| 5.4 At the end of the program, I feel that I could have used the robotic glove coupled to the virtual environment system without the help of a therapist given my level of competence with the glove and the system. |  |  | |  |  | |  |
| **6. Perceived health benefits** | | | | | | | |
| 6.1 At the end of the program, I noticed an improvement in muscle strength in the hand |  |  | |  |  | |  |
| 6.2 At the end of the program, I noticed an improvement in the control to close/open the hand |  |  | |  |  | |  |
| 6.3 At the end of the program, I noticed an improvement in control to use each finger separately |  |  | |  |  | |  |
| **7. Satisfaction with the attributes of the RAHRE Program** | | | | | | | |
| 7.1 The total duration of the program, which took place over a period of 2 weeks, was | **Adequate** | | **Too short** | | | **Too long** | |
| 7.2 The number of exercise sessions (5 times per week) is: | **Adequate** | | **Not enough** | | | **Too much** | |
| 7.3 The duration of each exercise session, which is approximately 30 min, is: | **Adequate** | | **Too short** | | | **Too long** | |
| 7.4 I perceived a level of physical exertion _______ during exercise sessions. | **Mild** | | **Moderate** | | | **High** | |
| 7.5 I perceived a level of cognitive effort (attention, concentration, etc.) _______ during exercise sessions. | **Mild** | | **Moderate** | | | **High** | |
